# The Prognostic Value of Circulating Tumor Cells in Asian Neuroendocrine Tumors

**DOI:** 10.1038/s41598-019-56539-z

**Published:** 2019-12-27

**Authors:** Jason Chia-Hsun Hsieh, Guan-Yu Chen, David Da-Wei Jhou, Wen-Chi Chou, Chun-Nan Yeh, Tsann-Long Hwang, Hung-Chi Lin, Hui-Chun Chu, Hung-Ming Wang, Tzu-Chen Yen, Jen-Shi Chen, Min-Hsien Wu

**Affiliations:** 1 Chang Gung University, Taoyuan, Taiwan; 2 Circulating Tumor Cells Lab, Division of Hematology-Oncology, Department of Internal Medicine, Chang Gung Memorial Hospital, Linkou, Taiwan; 3 Institute of Biomedical Engineering, College of Electrical and Computer Engineering, National Chiao Tung University, Hsinchu, Taiwan; 4 Department of Biological Science and Technology, National Chiao Tung University, Hsinchu, Taiwan; 5 Division of General Surgery, Department of Surgery, Chang Gung Memorial Hospital, Linkou, Taoyuan, Taiwan; 6 Molecular Imaging Center, Chang Gung Memorial Hospital, Linkou, Taoyuan, Taiwan; 7 Department of Nuclear Medicine, Linkou Chang Gung Memorial Hospital and Chang Gung University, Taoyuan, Taiwan; 8 Department of Chemical Engineering, Ming Chi University of Technology, New Taipei City, 24301 Taiwan; 9 Tissue Engineering and Microfluidic Biochip Lab, Graduate Institute of Biomedical Engineering, Chang Gung University, Taoyuan, Taiwan

**Keywords:** Prognostic markers, Neuroendocrine cancer

## Abstract

Circulating tumor cells (CTC) play important roles in various cancers; however, few studies have assessed their clinical utility in neuroendocrine tumors. This study aimed to prospectively evaluate the prognostic value of CTC counts in Asian patients with neuroendocrine tumors before and during anti-cancer therapy. Patients who were diagnosed with unresectable histological neuroendocrine tumors between September 2011 and September 2017 were enrolled. CTC testing was performed before and during anti-cancer therapy using a negative selection protocol. Chromogranin A levels were also assessed. Univariate and multivariate Cox’s proportional hazard model with forward LR model was performed to investigate the impact of independent factors on overall survival and progression-free survival. Kaplan–Meier method with log-rank tests were used to determine the difference among different clinicopathological signatures and CTC cutoff. The baseline CTC detection rate was 94.3% (33/35). CTC counts were associated with cancer stages (I-III vs. IV, *P* = 0.015), liver metastasis (*P* = 0.026), and neuroendocrine tumor grading (*P* = 0.03). The median progression-free survival and overall survivals were 12.3 and 30.4 months, respectively. In multivariate Cox regression model, neuroendocrine tumors grading and baseline CTC counts were both independent prognostic factors for progression-free survival (PFS, *P* = 0.005 and 0.015, respectively) and overall survival (OS, *P* = 0.018 and 0.023, respectively). In Kaplan-Meier analysis, lower baseline chromogranin A levels were associated with longer PFS (*P* = 0.024). Baseline CTC counts are associated with the clinicopathologic features of neuroendocrine tumors and are an independent prognostic factor for this malignancy.

## Introduction

Neuroendocrine tumors (NETs) can arise from neuroendocrine cells throughout the body and have heterogenous clinical manifestations. NETs are relatively rare, with an incidence of only approximately 5.25 per 100,000 population^1^. The treatments for NET comprise targeted therapies, including sunitinib^2^ and everolimus^3^; somatostatin analogues^4,5^; peptide receptor radionuclide therapy^6^; and chemotherapy^7^.

The prognosis of NET is commonly determined via tumor grading^8^ of primary cancer or metastatic tissues. However, the analyzed biopsy samples are often small and might not be representative of the whole tumor characteristics, particularly when the tumor is in the advanced stage or is highly aggressive^9^. Re-biopsy is recommended while the treatment of NET fails, but it is invasive and can be challenging to perform. Old cancer samples could not provide new information for subsequent therapies or current disease mechanisms.

A National Cancer Institute summit on NETs held in 2007 reported biomarker limitations to be a crucial factor yet to be addressed in the management of NETs^10^. Several circulating tumor markers have been used to capture real-time cancer information and predict treatment response and survival. Chromogranin A (CgA)^11^, chromogranin B^12^, and neuron-specific enolase^13^ have the potential to predict prognosis in patients with NET^11,14^.

Recently, liquid biopsies, a newer and wider-spectrum biomarker test, were also proposed to provide cytological, biological, morphological, and molecular information on cancer^15^. Liquid biopsies have been proven to be feasible to perform repeatedly to obtain information to monitor or guide NET therapies^16^.

Circulating tumor cells (CTCs), which are among the markers that can be evaluated via liquid biopsies, had been proposed to be a prognostic marker in various types of solid tumor, such as prostate, breast, and lung cancers^15^. CTCs are believed to be cells shed from primary/metastatic cancer mass and are thus closely related to the metastatic process of cancer^15,17,18^.

In NETs, CTCs identified via Cellsearch® have been proven to be prognostic for treatment response and survival^14,19^, although these CTC need to be validated further^10^. In literature, some investigators have published studies using negative enrichment techniques to identify or isolate CTCs considering CTC would possibly lose the expression of epithelial markers, such as epithelial cell adhesion molecule (EpCAM) or cytokeratins^20,21^. Several systems, such as EPISPOT^22^, ISET^23^ or flow cytometry-based systems^24^ were commonly used technologies in cancer researches. However, Cellsearch® is not available in many countries, and studies on CTCs identified via Cellsearch® were mainly conducted in the Caucasian population and NETs of gastrointestinal origin. The role of CTC in non-Caucasian NET patients and NET arising from other organs remain unclear.

Therefore, in this study, we aimed to prospectively evaluate whether CTC counts have a prognostic role in Asian patients with NET before and during anti-cancer therapy.

## Materials and Methods

### Patient selection

This study was designed as a prospective observational study at a single medical center. Patients who were histologically diagnosed with unresectable NET from any origin were enrolled. The study protocol was approved by the Institutional Review Board of Chang Gung Memorial Hospital, Linkou with approval numbers of 99–4095B, 100–4623 C, and 201601461B0. All methods were performed in accordance with the relevant guidelines and regulations. All enrolled patients signed informed consent after they were informed of the study design, scientific goals, and inconvenience/risks of participation. The other eligibility criteria included (a) age over 20 years and (b) measurable lesion confirmed via imaging studies before systemic treatment, including chemotherapy, targeted therapies or somatostatin analogues (SAAs).

All patients underwent a baseline evaluation that included demographic data, performance status (Eastern Cooperative Oncology Group Performance Score [ECOG-PS]), burden of liver metastasis if the patients had liver metastasis, treatment history, pathological characteristics, tumor differentiation, tumor grade determined via Ki-67% index according to the World Health Organization 2010 classification^8^, tumor stage according to the American Joint Committee on Cancer 7^th^ edition guidelines, computed tomography (CT) scan, and blood CgA test. Systemic anticancer therapy consisted of targeted therapies, chemotherapy, and SAAs, as decided by the physician based on the European Neuroendocrine Tumor Society, National Comprehensive Cancer Network, and local therapeutic guidelines. Tumor response was evaluated via CT scan according to the Response Evaluation Criteria in Solid Tumors 1.1^25^. Imaging studies were performed and interpreted by institutional radiologists at baseline and were repeated approximately every 12 weeks to evaluate tumor response. Overall survival (OS) and progression-free survival (PFS) were calculated from the date of CTC testing to the date of death and disease progression, respectively.

### Measurement of peripheral circulating tumor cells

CTC was measured within seven days of the first administration day of systemic treatments. Follow-up CTC tests were performed according to the physician’s judgment at any changes in disease status. CTC was identified using a protocol of combined negative selection and positive detection strategies, which was designed and validated in 2 previous studies^26,27^. In summary, CTC analysis comprised a two-step process as follows: (1) a negative selection protocol for effective red blood cell (RBC) and leukocyte depletion using lysis solutions and a CD45 depletion kit and (2) standard flow cytometry technique to quantitatively identify and calculate the number of CTCs.

Specifically, 8 mL of peripheral blood from each patient was used. The first 4 mL of blood was discarded to avoid epithelial contamination in CTC analysis. RBC lysis was performed within 24 hours after blood extraction. Samples were then negatively enriched by adding 25 μL/mL EasySep CD45 Depletion Cocktail (STEMCELL Technologies Inc., Vancouver, BC, Canada) and 50 μL/mL EasySep Magnetic Nanoparticles (STEMCELL). Immunomagnetically enriched samples containing spiked cancer cell lines (HCT116, a positive control cell line for Epithelial cell adhesion molecule [EpCAM], purchased from the Food Industry Research and Development Institute, Taiwan) were collected and labelled with Alexa Fluor® 488-conjugated anti-EpCAM mono-antibody (1:200 dilution; Cell Signalling Technology, Inc., Danvers, MA, USA.) and a fluorescent stain for labeling DNA, Hoechst 33,342 (1:500 in washing solution; Thermo Scientific, Waltham, MA, USA) for nuclear staining.

In addition, we routinely used 4 mL of peripheral blood drawn from healthy individuals that were spiked with and without 1,000 cancer cell lines for controls during the trial. The performance recovery rate, which was defined as the number of cancer cells identified via flow cytometry (Beckman Coulter Life Sciences, USA) divided by the number of spiked cancer cells, and the coefficient of variation value have been calculated and reported to be stable in a previous report^26^. CTCs were defined as the cells that were positive both for EpCAM and Hoechst after all processes (CD45^neg^). For routine quality control protocols in the laboratory, the investigators would calculate the number of CTCs under fluorescence microscopy to confirm the efficacy of CTC isolation and antibodies.

### Statistical analysis

The patients’ demographic data were summarized as the number (%) for categorical variables, and the median, 95% confidence interval (CI), and range for continuous variables. Chi-square test was used to assess the difference between basic patient characteristics and CTC count. Univariate and multivariate Cox’s proportional hazard model with forward LR model were performed to investigate the impact of independent factors on OS and PFS. Kaplan–Meier method with log-rank tests were used to determine the difference among different clinicopathological signatures and CTC numbers. Multiple testing and ROC curve methods were used to verify the cutoff values of CTCs even it might not be optimized for patients out of the study. All statistical analyses were 2-sided and performed using SPSS 18.0 software (SPSS Inc., Chicago, IL). A *P* value of < 0.05 was considered statistically significant.

## Results

### Patient characteristics

Between September 2011 and September 2017, 57 patients with histologically proven NET who received anti-cancer therapy were screened. Twenty-two patients who only received surgery were excluded for analysis due to no active following systemic anticancer therapy. Therefore, thirty-five patients who underwent systemic active anticancer therapy including somatostatin analogues were included for final analysis. The basic characteristics of the enrolled patients are shown in Table 1. Clinical information and survival data were updated until December 2018. The median age of the patients was 60 (range, 24–86) years. The primary sites of NET were gastrointestinal (n = 22, 62.9%), bronchopulmonary (n = 4, 11.4%), unknown primary (n = 4, 11.4%), head and neck (n = 2, 5.7%), thymic (n = 2, 5.7%), and skin (n = 1, 2.9%) origins. Regarding overall staging (AJCC 7^th^ edition), majority of patients had stage IV disease (n = 27, 77.1%), including locally advanced stage IV without distant metastasis, i.e., cancers originating from the head and neck area. The most common site of metastasis was the liver (n = 15, 68.2%) and lung (n = 7, 31.8%). Of the thirty-five patients, 11 (31.4%), 9 (25.7%), and 15 (42.9%) were categorized as grade I, II, and III tumors, respectively. Most patients had an ECOG PS of 0–1 (n = 29, 82.9%). Twenty patients (57.1%) had a baseline CgA of ≤120 ng/mL. The most common diagnosis status was newly diagnosed (n = 22, 61.9%) and R1 resection with recurrence (n = 8, 22.9%).

**Table 1:** Basic characteristics (n = 35).

	n	(%)
Age, median (range), years	60 (24–86)	
**Sex**		
Female/Male	12/23	34.3/65.7%
**Primary site of NET**		
Gastrointestinal tract origin	22	62.9%
Pancreas	11	31.4%
Colorectal	5	14.3%
Esophageal	2	5.7%
Gastric	2	5.7%
Gallbladder	1	2.9%
Small intestine	1	2.9%
Bronchopulmonary (lung and trachea)	4	11.4%
Unknown Primary	4	11.4%
Head and neck	2	5.7%
Thymus	2	5.7%
Skin (Merkel cell carcinoma)	1	2.9%
M0 status	11	31.4%
M1 status (with distant metastasis)	24	68.6%
**Metastatic sites**		
Liver	15	68.2%
Lung	7	31.8%
Lymph nodes	9	40.9%
Bone	4	18.2%
Brain	2	9.1%
Peritoneum	2	9.1%
Spleen	2	9.1%
**Tumor grade**		
1/2/3	11/9/15	31.4/25.7/42.9%
**ECOG PS**		
0–1	29	82.9%
>2	6	17.1%
**Baseline CgA, ng/mL**		
≤120	20	57.1%
>120	15	42.9%
**Baseline diagnosis status at enrollment**		
Newly diagnosed	22	62.9%
Post-surgery with recurrence	8	22.9%
PD on SSAs	3	8.6%
PD on palliative chemotherapy	1	2.9%
PD on SSAs + radioembolization	1	2.9%

*Abbreviations: CgA, chromogranin A; CTC, circulating tumor cell; ECOG PS, Eastern Cooperative Oncology Group Performance Status; PD: progressive disease; NET, neuroendocrine tumor; SSA: somatostatin analogue. *Some cancers are stage IV but have no distant metastasis, i.e., stage IVa and IVb in head and neck cancer.

### Correlation between baseline CTC count and clinicopathologic features

The detection rate of CTC count at baseline was 94.3% (33/35). The median baseline CTC count was 52.4 (range, 0.0 to 376.0; mean ± standard deviation, 93.4 ± 110.3). Table 2 shows the association between the clinical characteristics and CTC counts. CTC positivity at the cutoff value of 20.0 cells/mL was associated with distant metastasis (M0 vs. M1) (Chi-square *P* = 0.015). We defined the cutoff of CTC number primarily by multiple testing and ROC curves (Fig. S1). We used cancer-related death versus no death (alive) to see whether if CTC cutoffs can predict the events (cancer death) well or not, which demonstrates that a cutoff at 20.0 cells/mL could potentially predict cancer death in this limited cohort. At a CTC cutoff of 20.0 cells/mL, a higher CTC count was associated with a higher NET grade (Chi-square *P*-value = 0.03). Under the same cutoff, the CTC count was also associated with the existence of liver metastasis (Chi-square *P* = 0.026) and cancer death (*P* = 0.028). Meanwhile, we noticed no significant correlation between baseline CTCs and baseline blood CgA level (cutoff: 120 ng/mL).

**Table 2:** Correlation of circulating tumor cell counts to clinical outcomes.

Cutoffs of CTC number	20 cells/mL			5 cells/mL			1 cells/mL		
	≥20	<20	*P*	≥5	<5	*P*	≥1	<1	*P*
Female	9	3		9	3		11	1	
Male	17	6	0.944	18	5	0.827	23	0	0.343
M0 (n = 11)	5	6		5	6		10	1	
M1 (n = 24)	21	3	0.015^a^	22	2	0.006^a^	24	0	0.314
NET grading G1	5	6		6	5		10	1	
NET grading G2	8	1		8	1		9	0	
NET grading G3	13	2	0.030^a^	13	2	0.097	15	0	0.571
Liver metastasis (no)	12	8		12	8		19	1	
Liver metastasis (yes)	14	1	0.026^a^	15	0	0.005^a^	15	0	1.000
Baseline CgA < 120	14	4		14	4		18	0	
Baseline CgA ≥ 120	12	5	0.460	13	4	0.620	16	1	0.486
Alive at analysis	16	9		17	8		24	1	
Death at analysis	10	0	0.028^a^	10	0	0.042^a^	10	0	1.000

^a^The statistical significance was calculated using Fisher’s exact test. *Abbreviations: CTC, circulating tumor cells; NET, neuroendocrine tumor; CgA, chromogranin A.

### Univariate and multivariate analysis for independent factors

We then used univariate and multivariate cox proportional hazards regression (forward LR model) to identify potential independent prognostic factors (Table 3**)**. In multivariate analysis for disease progression, tumor grade showed an independent prognostic role (hazard ratio [HR]: 3.600; 95% confidence interval [CI], 1.475–8.787; *P* = 0.005). Tumor grade was also an independent prognostic factor for cancer death (HR: 6.195; 95% CI, 1.370–28.004; *P* = 0.018). After considering all possible confounding factors, baseline CTC counts had a strong association with survival. Baseline CTC counts were an independent prognostic factor for disease progression (HR: 1.006 for one CTC increase; 95% CI, 1.001–1.012; *P* = 0.015) and cancer death (HR: 1.009 for one CTC increase; 95% CI, 1.001–1.017; *P* = 0.023) in multivariate analysis.

**Table 3:** Univariate and multivariate analysis for survival impact of CTCs.

	PFS				OS			
	Univariate		Multivariate		Univariate		Multivariate	
	HR (95% CI)	*P* value	HR (95% CI)	*P* value	HR (95% CI)	*P* value	HR (95% CI)	*P* value
Age, years	1.045 (0.993–1.099)	0.089			1.089 (1.017–1.165)	0.014		
Sex (Male vs. Female)	1.540 (0.543–4.368)	0.417			6.067 (0.760–48.423)	0.089		
Staging	1.978 (0.839–4.662)	0.119			4.728 (0.250–89.341)	0.300		
NET grade	3.851 (1.637–9.062)	0.002	3.600 (1.475–8.787)	0.005	6.048 (1.509–24.242)	0.011	6.195 (1.370–28.004	0.018
Liver burden (%)	1.021 (0.996–1.047)	0.100			1.030 (0.998–1.064)	0.069		
ECOG PS	1.238 (0.747–2.053)	0.407			1.751 (0.963–3.182)	0.066		
Prior surgery (yes vs no)	0.462 (0.150–1.422)	0.178			0.019 (0.000–2.612)	0.115		
Lung metastasis (yes vs. no)	1.943 (0.710–5.312)	0.196			2.066 (0.579–7.379)	0.264		
Liver metastasis (yes vs. no)	1.534 (0.589–3.996)	0.381			1.030 (0.998–1.064)	0.069		
Baseline CgA ≥ 120	0.853 (0.337–2.159)	0.738			2.426 (0.618–9.517)	0.204		
Baseline CTC (cells/mL)	1.008 (1.003–1.013)	0.002	1.006 (1.001–1.012)	0.015	1.011 (1.003–1.019)	0.005	1.009 (1.001–1.017)	0.023

*Abbreviations: NET, neuroendocrine tumor; ECOG PS, Eastern Cooperative Oncology Group Performance Status; CgA, chromogranin A; CTC, circulating tumor cells; PFS, progression-free survival; OS, overall survival; HR, hazard ratio; CI, confidence interval.

### Kaplan-Meier curves for survival impact

**Figure 1:**
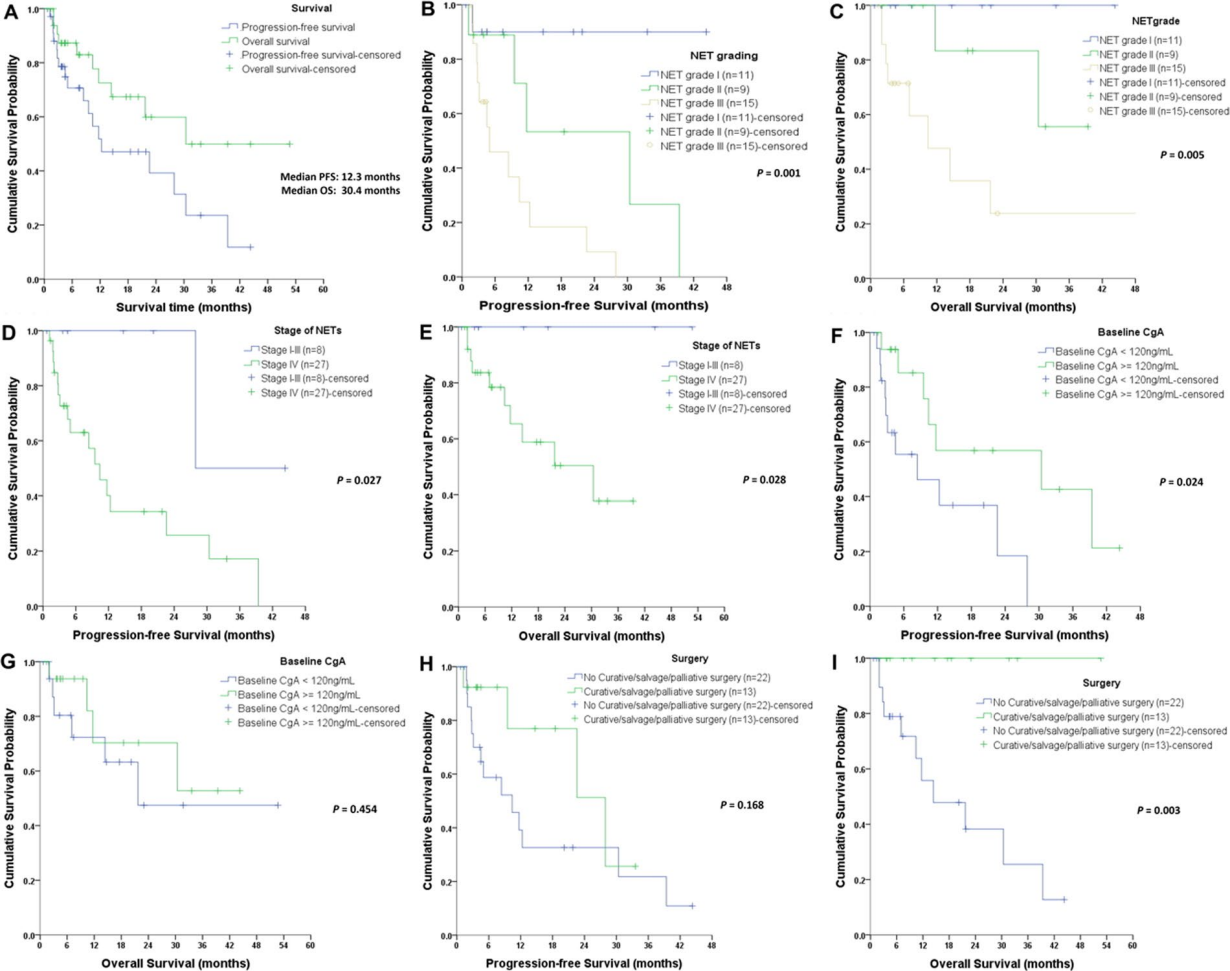
Kaplan-Meier curves of progression-free survival (PFS) and overall survival (OS) according to clinicopathologic features. (**A**) Median PFS and OS of the whole study population. **(B**,**C)** show that higher NET grade (World Health Organization 2010 edition) correlate shorter PFS and OS. **(D**,**E)** demonstrate that higher tumor stages (American Joint Cancer Committee [AJCC] 7^th^ edition) correlate to shorter PFS and OS. **(F)** shows that higher baseline blood chromogranin A level indicates a superior PFS, but it is not significant for OS **(G)**. **(I)** shows an OS benefit from curative and palliative surgery, whereas no PFS benefits are noted in **(H)**.

The median (±standard error, SE) PFS of the entire group was 12.3 ± 7.2 months, and the median (±SE) OS was 30.4 ± 8.7 months (Fig. 1A). NET grade was associated with PFS and OS (*P* = 0.001 and 0.005, respectively; Fig. 1B,C). Compared to NET patients with stage IV (n = 27), those with stage I-III (n = 8) had a better PFS (Fig. 1D, *P* = 0.027) and OS (Fig. 1E, *P* = 0.028). Baseline blood CgA level ≥120 ng/mL was associated with a longer PFS (*P* = 0.024, Fig. 1F), but was not associated with OS (*P* = 0.454, Fig. 1G). The blood CgA was further proven not significant in multivariate analyses (Table 3). Surgery was beneficial for OS, but not for PFS, inpatients who underwent palliative (debulking), curative, or salvage surgery (*P* = 0.003, Fig. 1I) (*P* = 0.168, Fig. 1H).

**Figure 2:**
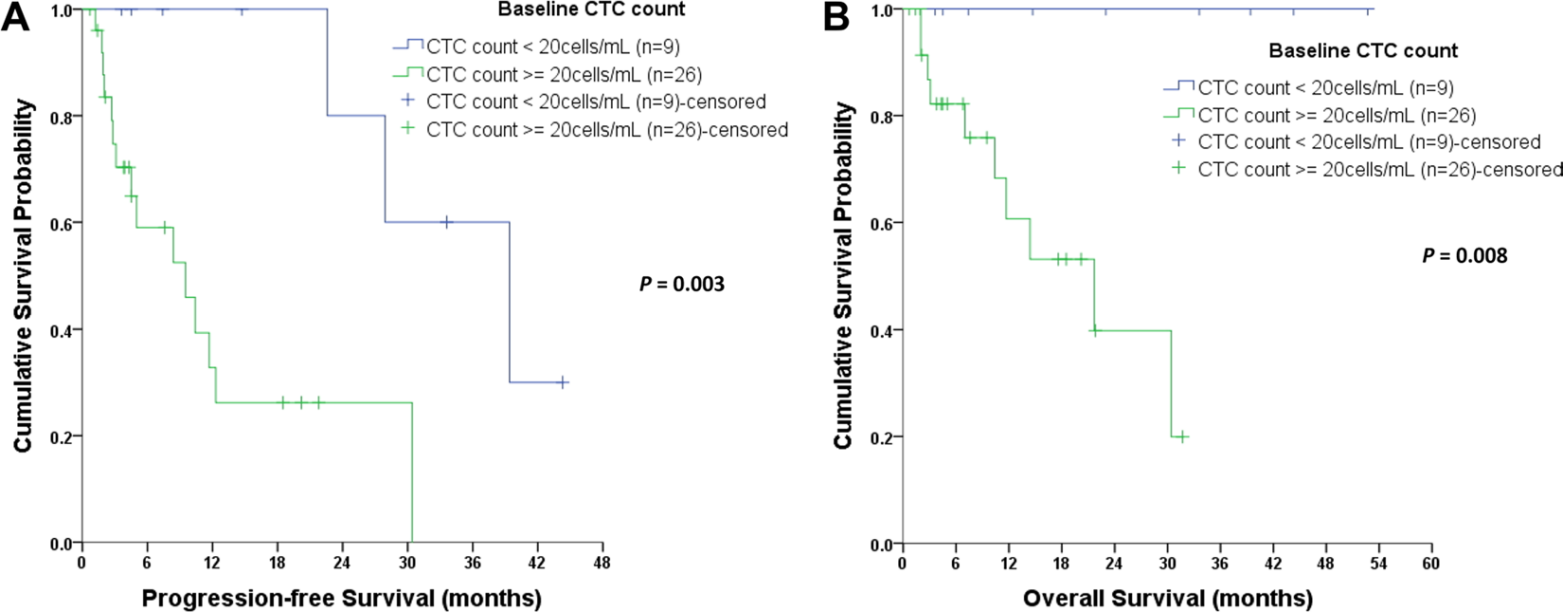
Kaplan-Meier curves of progression-free survival (PFS) and overall survival (OS) according to circulating tumor cells (CTC). (**A**,**B)** show that lower baseline CTCs (<20 cells/mL of blood) could both predict a better PFS and OS.

**Figure 3:**
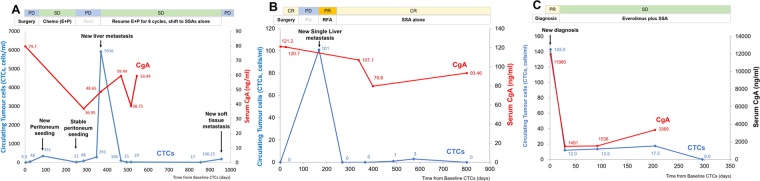
Longitudinal changes of circulating tumor cell (CTC) count and their correlations to treatment response, cancer progression, and chromogranin A (CgA) levels in three individual cases. (**A)** Patient #19 was a 65-year-old woman diagnosed with grade 3 pancreatic neuroendocrine carcinoma (well-differentiated; Ki-67 index, 30%; and mitotic index, 10/20 *high*-*power field*) with peritoneal seeding since February in 2015. After palliative resection for pancreatic lesions, she started to receive palliative etoposide plus cisplatin after the surgery. Her baseline CTC count was 4.3 cells/ml of blood. CTC count was highly correlated with the clinical course of the disease. **(B)** A 55-year-old man with grade I pancreatic neuroendocrine tumor with hepatic metastasis (Patient # 22). He underwent major curative surgery and developed one episode of hepatic recurrence. CTC was substantially elevated at recurrence and decreased rapidly after radio-frequent ablation for liver lesion, whereas blood chromogranin A (CgA) did not decrease. The patient was clinically disease free until the last follow-up. (**C**) The clinical course of a patient with grade II pancreatic neuroendocrine tumor with multiple hepatic metastases. Changes in CgA and CTC levels were highly correlated to clinical events.

Regarding baseline CTCs at enrollment (before systemic treatment), CTCs < 20 cells/mL were found to be associated with a longer PFS (*P* = 0.003, Fig. 2A) and OS (*P* = 0.008, Fig. 2B). In the 28 (80.0%) patients who underwent follow-up CTC tests, the longitudinal trend of CTC count was found to be highly correlated to cancer status during treatment. We performed a total of 116 CTC tests in this cohort. A decrease in CTC count within three months of treatment was associated with disease status (*P* = 0.022, data not shown). The CTC count, CgA level, and disease status of three representative patients are shown in Fig. 3A–C.

## Discussion

In the present study, we found that baseline CTC count was associated with cancer stage (stage I–III vs. IV), the existence of liver metastasis, NET grade, and cancer death. These findings were consistent with those obtained by Khan *et al*.^28^, who reported a correlation between CTC levels and liver metastases. In their subsequent study published in 2013^19^, the presence of CTC was associated with increased tumor grade. We also found that both NET grade and baseline CTC counts were independent prognostic factors for NET progression and cancer death. Furthermore, during longitudinal CTC follow-up in our study, changes in CTC counts were found to be highly associated with treatment responses, indicating that CTC testing might be used for monitoring cancer status. These results are also consistent with those of studies on Merkel cell carcinoma^29,30^ and gastrointestinal and pulmonary NET^14^. The differences among similar studies addressing the role of CTCs in NET patients are summarized in Table 4. Compared with other studies analyzing CTCs in small-cell lung cancer (SCLC)^31,32^, our study reported an overall decisive role of CTCs in NET patients originating from various primary sites. However, some findings were in contrast to those in our study. One study reported that the consensus among healthcare providers is that CTCs are not a reliable biomarker in NET because of validation issues^10^. In this consensus, only 2 CTC-related articles were reviewed, and clinicians often conservatively refused to accept new diagnostic tools possibly owing to a lack of information and experience. CTCs are live cancer cells obtained from living cancer patients, and they are thus more reliable than other biomarkers. Therefore, CTC remains one of the most promising biomarkers for investigating cancer progression, metastasis, and evolution in basic and translational NET research.

**Table 4:** Summary of studies on the role of circulating tumor cell in neuroendocrine tumors.

Author	Nation	No. of patients	Cancer type	Cancer stages	CTC positive detection rate	Strategy	Methods	CTC follow-up	Major results
**Caucasian population**									
^28^	UK	79	GI and Pulmonary NETs	Metastatic	35.1% (≥1)	Positive	CellSearch	No	The absence of CTCs was strongly associated with stable disease. CTC levels are correlated with urinary 5-HIAA and burden of liver metastases.
^19^	UK	175	GI and Pulmonary NETs	Metastatic	49.1% (≥1), 30% (≥5)	Positive	CellSearch	No	Training set and validation set. CTCs were associated with increased burden, increased tumor grade, elevated blood CgA, and independent prognostic factor of worse PFS and OS.
^29^	USA	34	Skin Meckel cell carcinoma	All stages	40% (≥1)	Positive	CellSearch	Yes	CTC played a prognostic role in patients with regional nodal disease.
^30^	Germany	30	Skin Meckel cell carcinoma	All stages	97% (≥0)	Positive	Laser Scanning Cytometry	Yes	CTC counts were elevated in patients with active disease. NSE and CgA blood levels did not correlate with PFS, DFS, OS, or recurrence.
^49^	USA	12	Prostate cancer with neuroendocrine phenotype	Metastatic	46.1% (≥5)	Positive	CellSearch	No	CTCs from NEPC patients demonstrated frequent clusters, low or absent androgen receptor expression, lower cytokeratin expression, and smaller morphology relative to typical CRPC.
^14^	UK	138	GI and Pulmonary NETs	Metastatic	60%( ≥ 1)	Positive	CellSearch	Yes	Changes in CTCs and baseline zero CTC count were strongly associated with OS.
**Asian population**									
Current work, 2019	Taiwan	35	GI, pulmonary, thymic, skin, head and neck	All stages	97.1% of baseline CTC	Negative	Negative selection + flow cytometry	Yes	Baseline CTCs are associated with tumor stage, tumor grade, liver metastasis, PFS, and OS. NET grading and baseline CTC were independent prognostic factors.

The optimal cutoff value of CTC remains challenging because the CTC counts vary significantly depending on the protocol, device, or strategy of CTC isolation^33^. Our study determined the CTC count using a negative selection strategy (EpCAM independent) that is believed to be capable of separating more suspicious cancer cells that are EpCAM^neg^ or EpCAM^lo^ (i.e., EpCAM^neg^-CTCs)^34,35^. The CTC count and detection rates of an EpCAM-independent (negative selection) method have been known to be generally higher than those in an EpCAM-dependent (positive selection) method, i.e., Cellsearch^®^. In the literature, a CTC cutoff of ≥1 cells/7.5 mL blood using Cellsearch^®^ has been proposed and validated to be clinically significant in NET^14,19,28^. Meanwhile, a cutoff of ≥5 cells/7.5 mL blood is more frequently used in other cancer types^36,37^. In the present study, we have tested multiple cutoff values of CTC counts, and three cutoffs (≥1.0^38,39^, ≥5.0^39^, ≥20.0^40^ cells/mL blood) with reported clinical significance in the literature were found to be significant in the association with clinicopathological characteristics (Table 2 and Fig. S1). The result showed a similar trend of a higher CTC level indicating a more severe clinical condition, including staging and distant metastasis. According to ROC and area under curves, we, therefore, set our cutoff value at 20.0 cells/mL blood. Although this is indeed the first study to use a negative-selection strategy (Table 4**)** in patients with neuroendocrine tumor, we are not proposing 20.0 cells/mL blood as an optimal cutoff as the sample size in this study was relatively small. Further large-scale prospective trials are still warranted to validate the significance of the cutoff.

CgA has been considered as a useful biomarker for the diagnosis and prognosis of patients with NET^41^. Our results showed that higher CgA levels indicated a superior PFS but not significant in multivariate analysis (Fig. 1F and Table 3). One of the reasons for these results might come from small sample size and other confounding factors. Meanwhile, our results on the correlations between CgA and CTCs were inconsistent with those of previous studies (Fig. 3). One study reported the difference in prognostic value between CgA and CTCs^28^. Gaiser *et al*. observed a correlation between CTCs and survival, but not between CgA and survival, in Meckel cell carcinoma^30^. Furthermore, Kahn *et al*. revised their previous findings in 2013^19^ and reported that changes in CgA were not significantly associated with survival^14^. Our findings suggest that CTCs might be more sensitive to disease change than CgA; however, further prospective comparative studies are still needed before a solid conclusion can be reached.

An OS benefit from aggressive surgery was observed in our cohorts (Figure H,I). Similar results were also reported in patients with localized pancreatic NETs ≤ 2 cm^42^. Those with N1 pancreatic NET^43^, small intestine NET^44^, unresectable carcinoid tumors^45^ and even those with peritoneal carcinomatosis^46^ obtained an OS benefit from aggressive surgery. However, some researchers reported conflicting results. One study reported that an upfront surgery in asymptomatic patients with stage IV small intestine NET did not provide survival benefits^47^. Our study included 2 cases of NET arising from the head and neck region. One had stage III disease treated via CCRT, and the other had multiple metastatic lesions and soon died because of poor response to systemic chemotherapy. This information might be valuable because head and neck NET is rarer than NETs arising from the gastrointestinal tract or pulmonary sites.

There are several limitations to our report. First, the relatively small sample size partially limits the strength of the findings. The small size is primarily due to the single-center design and the rare incidence of the cancer^48^. Also, the overall number of patients and the incidence of NET in our area is relatively small (1.51/100,000)^48^. Despite these limitations, we believe that this study is still essential because this is the only study elucidating the prognostic role of CTCs in NET patients in Asia. Second, although the study enrolled patients with different primary sites of NET to perform a detailed analysis, the possible role of CTCs in the different primary site of NETs could be underestimated because of the small sample size. Further large-scale studies focusing on a single primary site are required to determine the value of CTCs in one single type of NET. However, almost all current literature enrolled patients with various primary sites of NET^14,19,28–30,49^, Table 4). Third, the staging of NET mainly depends on the primary sites, and the prognosis of each stage differs. For example, a patient with stage IVa head and neck NET with grade 3 differentiation can be possibly cured via cisplatin-based concurrent chemoradiotherapy (CCRT). The overall prognosis is considerably different in those with a pancreatic grade III NET, which possibly contributed some bias in the analysis. Fourth, Cellsearch® was not used to identify and evaluate CTC primarily because it is relatively expensive and device-dependent with relatively low CTC detection rates even in patients with metastatic cancer^50^. We proposed an economical, easy-to-perform, and commonly available isolation/identification method with higher detection rates and validated in many kinds of cancer populations^26,51,52^. This method is feasible in most regular laboratories.

In conclusion, our study found that baseline CTC counts are associated with the clinicopathologic features of NET and could be an independent prognostic factor for survival. In addition to CgA, CTC might also be a useful biomarker for determining the prognosis of patients with NET. Our findings should be validated in large-scale prospective clinical trials.

## Supplementary information

Supplementary figure S1, Supplementary figure S2

## Data Availability

All data generated or analysed during this study are included in this published article (and its Supplementary Information Files).

## References

[1] Yao JC (2008). One hundred years after “carcinoid”: epidemiology of and prognostic factors for neuroendocrine tumors in 35,825 cases in the United States. Journal of Clinical Oncology.

[2] Raymond E (2011). Sunitinib malate for the treatment of pancreatic neuroendocrine tumors. New England Journal of Medicine.

[3] Yao James C., Pavel Marianne, Lombard-Bohas Catherine, Van Cutsem Eric, Voi Maurizio, Brandt Ulrike, He Wei, Chen David, Capdevila Jaume, de Vries Elisabeth G.E., Tomassetti Paola, Hobday Timothy, Pommier Rodney, Öberg Kjell (2016). Everolimus for the Treatment of Advanced Pancreatic Neuroendocrine Tumors: Overall Survival and Circulating Biomarkers From the Randomized, Phase III RADIANT-3 Study. Journal of Clinical Oncology.

[4] Rinke A (2009). Placebo-controlled, double-blind, prospective, randomized study on the effect of octreotide LAR in the control of tumor growth in patients with metastatic neuroendocrine midgut tumors: a report from the PROMID Study Group. Journal of Clinical Oncology.

[5] Caplin ME (2014). Lanreotide in metastatic enteropancreatic neuroendocrine tumors. New England Journal of Medicine.

[6] Bodei L (2016). Measurement of circulating transcripts and gene cluster analysis predicts and defines therapeutic efficacy of peptide receptor radionuclide therapy (PRRT) in neuroendocrine tumors. Eur J Nucl Med Mol Imaging.

[7] Garcia-Carbonero R (2017). ENETS Consensus Guidelines for the Standards of Care in Neuroendocrine Neoplasms: Systemic Therapy-Chemotherapy. Neuroendocrinology.

[8] Bosman, F. T., Carneiro, F., Hruban, R. H. & Theise, N. D. *WHO classification of tumours of the digestive system*. (World Health Organization, 2010).

[9] Gerlinger M (2012). Intratumor heterogeneity and branched evolution revealed by multiregion sequencing. N Engl J Med.

[10] Oberg K (2015). Consensus on biomarkers for neuroendocrine tumour disease. The Lancet Oncology.

[11] Chou W-C (2014). Plasma chromogranin A levels predict survival and tumor response in patients with advanced gastroenteropancreatic neuroendocrine tumors. Anticancer research.

[12] Ramachandran R (2015). Comparison of the utility of Cocaine-and Amphetamine-Regulated Transcript (CART), chromogranin A, and chromogranin B in neuroendocrine tumor diagnosis and assessment of disease progression. *The*. Journal of Clinical Endocrinology & Metabolism.

[13] Korse CM (2012). Choice of tumour markers in patients with neuroendocrine tumours is dependent on the histological grade. A marker study of Chromogranin A, Neuron specific enolase, Progastrin-releasing peptide and cytokeratin fragments. European journal of cancer.

[14] Khan MS (2016). Early Changes in Circulating Tumor Cells Are Associated with Response and Survival Following Treatment of Metastatic Neuroendocrine Neoplasms. Clin Cancer Res.

[15] Pantel K, Speicher M (2016). The biology of circulating tumor cells. Oncogene.

[16] Kidd M, Modlin IM (2017). Therapy: The role of liquid biopsies to manage and predict PRRT for NETs. Nature Reviews Gastroenterology and Hepatology.

[17] Massagué J, Obenauf AC (2016). Metastatic colonization by circulating tumour cells. Nature.

[18] Aceto N, Toner M, Maheswaran S, Haber DA (2015). En route to metastasis: circulating tumor cell clusters and epithelial-to-mesenchymal transition. Trends in Cancer.

[19] Khan MS (2013). Circulating tumor cells as prognostic markers in neuroendocrine tumors. J Clin Oncol.

[20] Hsieh, J. C. H. & Wu, T. M. H. The selection strategy for circulating tumor cells (CTCs) isolation and enumeration: technical features, methods and clinical applications. *Tumor Metastasis* (2016).

[21] Ozkumur E (2013). Inertial focusing for tumor antigen–dependent and–independent sorting of rare circulating tumor cells. Science translational medicine.

[22] Alix-Panabières, C. *In* Minimal Residual Disease and Circulating Tumor Cells in Breast Cancer 69–76 (Springer, 2012).

[23] Farace F (2011). A direct comparison of CellSearch and ISET for circulating tumour-cell detection in patients with metastatic carcinomas. British journal of cancer.

[24] Lu Y (2015). Isolation and characterization of living circulating tumor cells in patients by immunomagnetic negative enrichment coupled with flow cytometry. Cancer.

[25] Eisenhauer EA (2009). New response evaluation criteria in solid tumours: revised RECIST guideline (version 1.1). European journal of cancer.

[26] Su PJ (2016). Circulating Tumour Cells as an Independent Prognostic Factor in Patients with Advanced Oesophageal Squamous Cell Carcinoma Undergoing Chemoradiotherapy. Scientific Reports.

[27] Chiu TK (2016). Application of optically-induced-dielectrophoresis in microfluidic system for purification of circulating tumour cells for gene expression analysis-Cancer cell line model. Scientific Reports.

[28] Khan MS (2011). Circulating tumor cells and EpCAM expression in neuroendocrine tumors. Clin Cancer Res.

[29] Blom A (2014). Clinical utility of a circulating tumor cell assay in Merkel cell carcinoma. J Am Acad Dermatol.

[30] Gaiser MR (2015). Evaluating blood levels of neuron specific enolase, chromogranin A, and circulating tumor cells as Merkel cell carcinoma biomarkers. Oncotarget.

[31] Naito T (2012). Prognostic impact of circulating tumor cells in patients with small cell lung cancer. Journal of Thoracic Oncology.

[32] Igawa S (2014). Circulating tumor cells as a prognostic factor in patients with small cell lung cancer. Oncology letters.

[33] Hsieh, J. C. H. & Wu, T. M. H. *In* Tumor Metastasis (InTech, 2016).

[34] Liu Z (2011). Negative enrichment by immunomagnetic nanobeads for unbiased characterization of circulating tumor cells from peripheral blood of cancer patients. J Transl Med.

[35] Xu Y (2017). Circulating tumor cell detection: A direct comparison between negative and unbiased enrichment in lung cancer. Oncology letters.

[36] Cristofanilli M (2004). Circulating tumor cells, disease progression, and survival in metastatic breast cancer. New England Journal of Medicine.

[37] De Bono JS (2008). Circulating tumor cells predict survival benefit from treatment in metastatic castration-resistant prostate cancer. Clinical cancer research.

[38] Alvarez Cubero MJ (2017). Circulating Tumor Cells: Markers and Methodologies for Enrichment and Detection. Methods Mol Biol.

[39] Wang SS (2019). Direct Plasmon-Enhanced Electrochemistry for Enabling Ultrasensitive and Label-Free Detection of Circulating Tumor Cells in Blood. Anal Chem.

[40] Weng WH (2018). Real-time circulating tumor cells detection via highly sensitive needle-like cytosensor-demonstrated by a blood flow simulation. Biosens Bioelectron.

[41] Yao JC (2011). Chromogranin A and Neuron-Specific Enolase as Prognostic Markers in Patients with Advanced pNET Treated with Everolimus. J Clin Endocr Metab.

[42] Sharpe SM, In H, Winchester DJ, Talamonti MS, Baker MS (2015). Surgical Resection Provides an Overall Survival Benefit for Patients with Small Pancreatic Neuroendocrine Tumors. Journal of Gastrointestinal Surgery.

[43] Tao L (2017). Surgical resection of primary tumor improves survival of pancreatic neuroendocrine tumor with liver metastases. Oncotarget.

[44] Norlén O (2012). Long-term results of surgery for small intestinal neuroendocrine tumors at a tertiary referral center. World journal of surgery.

[45] Gulec SA, Mountcastle TS, Frey D, Cundiff JD (2002). Cytoreductive surgery in patients with advanced-stage carcinoid tumors/Discussion. The American Surgeon.

[46] De Mestier L (2015). Updating the surgical management of peritoneal carcinomatosis in patients with neuroendocrine tumors. Neuroendocrinology.

[47] Daskalakis, K. *et al*. Association of a Prophylactic Surgical Approach to Stage IV Small Intestinal Neuroendocrine Tumors With Survival. *JAMA oncology* (2017). 10.1001/jamaoncol.2017.3326 PMC5838704 29049611

[48] Tsai HJ (2013). The epidemiology of neuroendocrine tumors in Taiwan: a nation-wide cancer registry-based study. PLoS One.

[49] Beltran H (2016). The Initial Detection and Partial Characterization of Circulating Tumor Cells in Neuroendocrine Prostate Cancer. Clin Cancer Res.

[50] Andreopoulou E (2012). Comparison of assay methods for detection of circulating tumor cells in metastatic breast cancer: AdnaGen AdnaTest BreastCancer Select/Detect™ versus Veridex CellSearch™ system. International journal of cancer.

[51] Chou W-C (2018). A Prognostic Model Based on Circulating Tumour Cells is Useful for Identifying the Poorest Survival Outcome in Patients with Metastatic Colorectal Cancer. International journal of biological sciences.

[52] Hsieh JCH (2015). Prognostic value of circulating tumor cells with podoplanin expression in patients with locally advanced or metastatic head and neck squamous cell carcinoma. Head & neck.

