# Supplementary material for: The Prognostic Value of Circulating Tumor Cells in Asian Neuroendocrine Tumors
Source: Sci Rep. 2019 Dec 27;9:19917. doi: 10.1038/s41598-019-56539-z (PMC6934482; doi:10.1038/s41598-019-56539-z)
Supplement: Supplementary file 1 — Supplementary figure S1, Supplementary figure S2 [file 41598_2019_56539_MOESM1_ESM.docx]

**Supplementary Figures**

**Type:** Original article

**Article Title:**

**The Prognostic Value of Circulating Tumor Cells in Asian Neuroendocrine Tumors**

**Authors:** Jason Chia-Hsun Hsieh^1,2^, M.D. Ph.D. Guan-Yu Chen^3,4^ Ph.D., David Da-Wei Jhou^1,2^ M.D, Wen-Chi Chou^1,2^ M.D. Ph.D., Chun-Nan Yeh^1,5^ M.D. Tsann-Long Hwang^1,5^ M.D., Hung-Chi Lin^1,2^ Ph.D., Hui-Chun Chu^1,2^ Ph.D. Hung-Ming Wang^1,2^ M.D., Tzu-Chen Yen^1,6,7^ M.D. Ph.D. Jen-Shi Chen^1,2^† M.D., and Min-Hsien Wu^1,2,8,9^† Ph.D.,

**Legend**

**Figure S1. Multiple ROC suggests a cutoff at 20.0 cells/mL in this cohort.** The ROC curve calculated the area under the curve to predict cancer-related death. We used cancer-related death versus no death (alive) to see whether if CTC cutoffs can predict the events (cancer death) well or not, which demonstrates that a cutoff at 20.0 cells/mL could potentially predict cancer death in this limited cohort.

**Figure S2. Circulating tumor cells across different primary site.** However, the difference was not statistically significant.

| 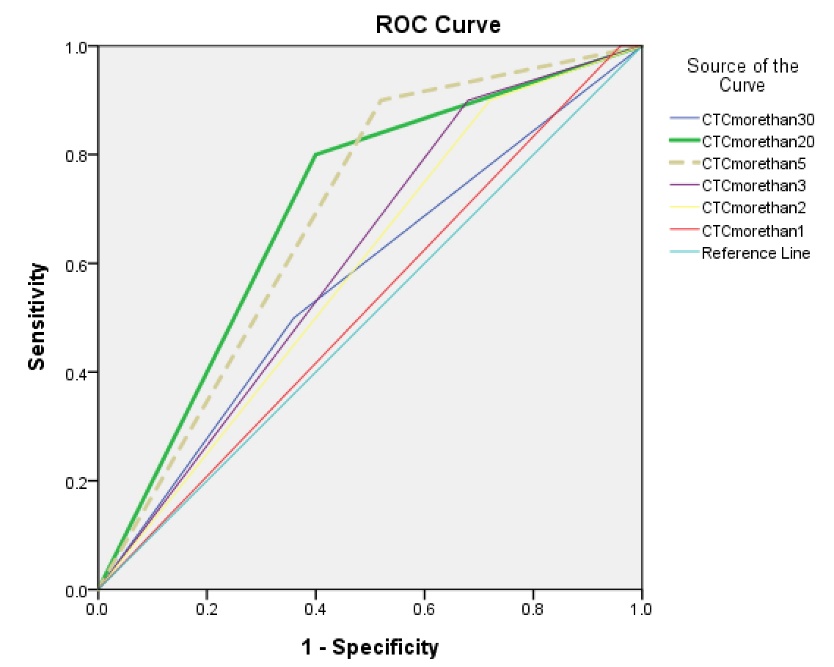 |
| --- |
| 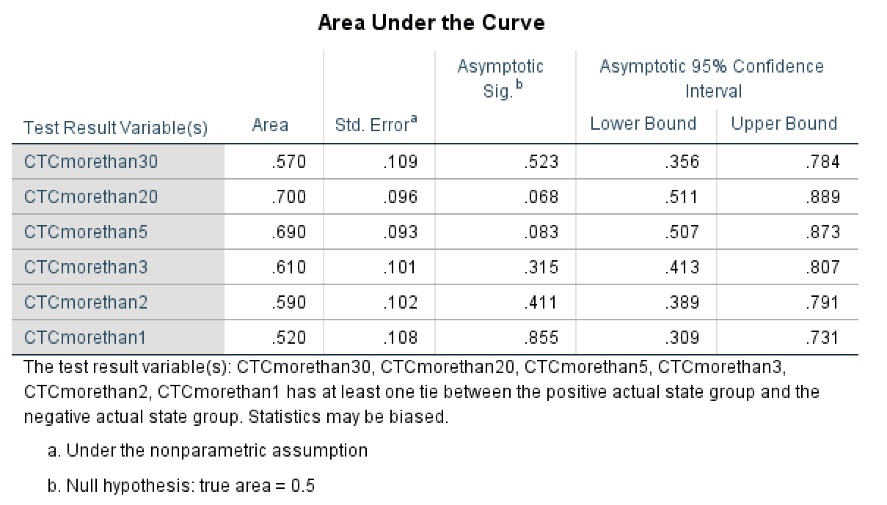 |

Figure S1. Multiple ROC suggests a cutoff at 20.0 cells/mL in this cohort.


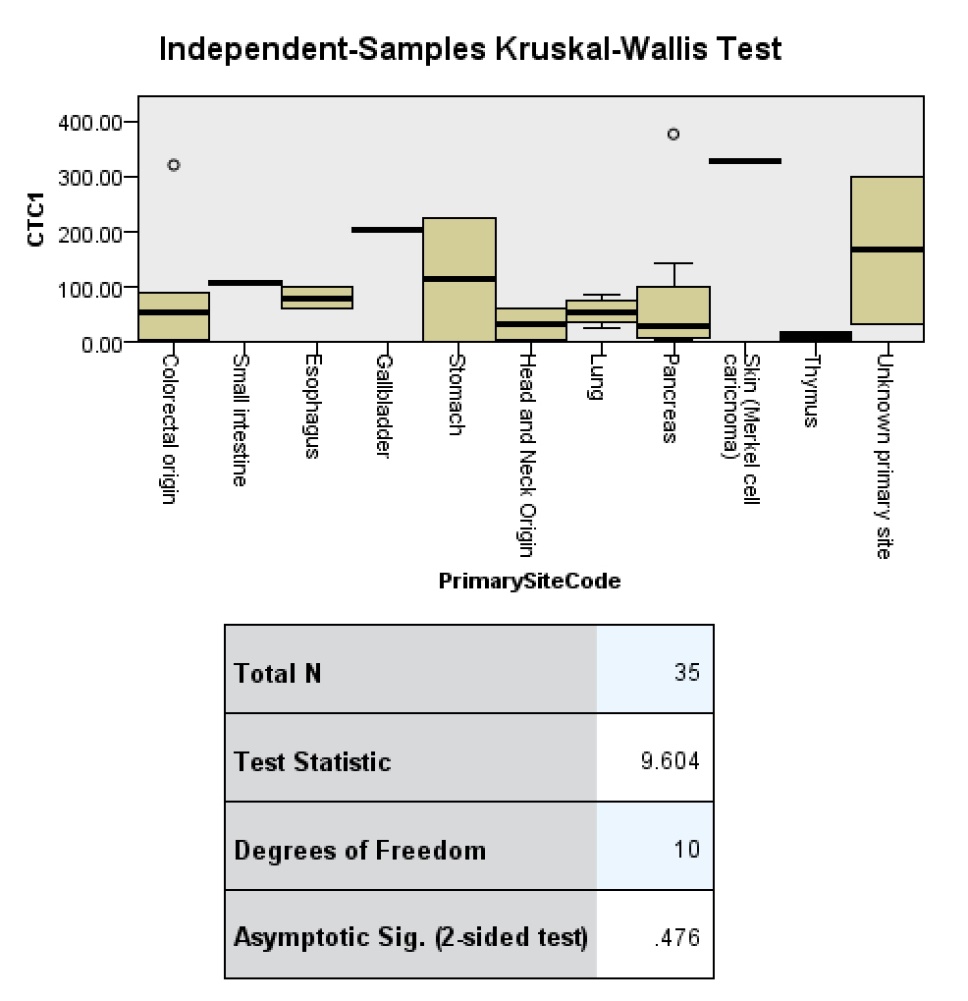


Figure S2. Circulating tumor cells across different tumor primary sites.
